# Sulforaphane Restores Cellular Glutathione Levels and Reduces Chronic Periodontitis Neutrophil Hyperactivity *In Vitro*

**DOI:** 10.1371/journal.pone.0066407

**Published:** 2013-06-24

**Authors:** Irundika H. K. Dias, Ian L. C. Chapple, Mike Milward, Melissa M. Grant, Eric Hill, James Brown, Helen R. Griffiths

**Affiliations:** 1 Life and Health Sciences, Aston Research Centre for Healthy Ageing, Aston University, Birmingham, United Kingdom; 2 School of Dentistry, College of Medical & Dental Sciences, University of Birmingham, Birmingham, United Kingdom; University of Southern California, United States of America

## Abstract

The production of high levels of reactive oxygen species by neutrophils is associated with the local and systemic destructive phenotype found in the chronic inflammatory disease periodontitis. In the present study, we investigated the ability of sulforaphane (SFN) to restore cellular glutathione levels and reduce the hyperactivity of circulating neutrophils associated with chronic periodontitis. Using differentiated HL60 cells as a neutrophil model, here we show that generation of extracellular O_2_^. -^ by the nicotinamide adenine dinucleotide (NADPH) oxidase complex is increased by intracellular glutathione depletion. This may be attributed to the upregulation of thiol regulated acid sphingomyelinase driven lipid raft formation. Intracellular glutathione was also lower in primary neutrophils from periodontitis patients and, consistent with our previous findings, patients neutrophils were hyper-reactive to stimuli. The activity of nuclear factor erythroid-2-related factor 2 (Nrf2), a master regulator of the antioxidant response, is impaired in circulating neutrophils from chronic periodontitis patients. Although patients’ neutrophils exhibit a low reduced glutathione (GSH)/oxidised glutathione (GSSG) ratio and a higher total Nrf2 level, the DNA-binding activity of nuclear Nrf2 remained unchanged relative to healthy controls and had reduced expression of glutamate cysteine ligase catalytic (GCLC), and modifier (GCLM) subunit mRNAs, compared to periodontally healthy subjects neutrophils. Pre-treatment with SFN increased expression of GCLC and GCM, improved intracellular GSH/GSSG ratios and reduced agonist-activated extracellular O_2_^. -^ production in both dHL60 and primary neutrophils from patients with periodontitis and controls. These findings suggest that a deficiency in Nrf2-dependent pathways may underpin susceptibility to hyper-reactivity in circulating primary neutrophils during chronic periodontitis.

## Introduction

Periodontitis is a ubiquitous chronic inflammatory disease initiated by a microbial biofilm, and in which a dysregulated immune-inflammatory response leads to destruction of the supporting tissues of the tooth and ultimately tooth loss. Apart from the damage caused locally to the periodontium, periodontitis is also recognised as a significant risk factor for atherogenic vascular disease [1] and type II diabetes [2]. There is evidence that oxidative stress is one of the key factors explaining some of the systemic pathophysiological mechanisms associated with inflammatory conditions such as periodontitis [3].

Although the underlying mechanisms of chronic periodontitis remain unclear, peripheral blood neutrophil hyper-activity (un-stimulated cells) and -reactivity (to a stimulus) is a key feature of the disease [4,5]. Reactive oxygen species (ROS) production by neutrophils during the respiratory burst is important in clearing local periodontal pathogens but is thought to be a significant factor in the aetiology of local tissue damage [3,6]. It has been previously shown that gingival crevicular fluid which protects the periodontal tissues, has less buffering capacity against ROS in periodontitis patients than in healthy controls [7]; the reduced glutathione (GSH) concentration is lower in the extracellular environment in periodontitis. It is likely that this is a consequence of increased ROS production by neutrophils in periodontitis patients.

The generation of the superoxide anion radical via nicotinamide adenine dinucleotide phosphate (NADPH) oxidase is the first step in the production of a range of reactive oxygen species. Although superoxide (O_2_^.-^) initiated cascades of reactions are important in clearing foreign pathogens, largely within the safe confines of the phagocytic vacuole, sustained NADPH oxidase (NOX) activation is considered to result in adverse effects on the host [3]. This is antagonised by cellular systems which include naturally occurring enzymes e.g. catalase, superoxide dismutase, glutathione (GSH) peroxidase, thioredoxin reductase and smaller molecular peptides and proteins e.g. intracellular glutathione and thioredoxin, which prevent the uncontrolled formation of free radicals and other reactive oxygen species. Among these, glutathione (GSH) plays a pivotal role in cellular redox homeostasis [8].

Redox regulation of gene expression and activity is described for many proteins [9] and enzymes that undergo reversible thiol oxidation [10]. Acid sphingomyelinase (ASMase) is indirectly activated in this way and its activation correlates with its translocation from intracellular stores onto the extracellular leaflet of the cell membrane [11]. SMase hydrolyzes sphingomyelin to ceramide and phosphocholine [11,12]. It has been shown that ceramide is incorporated to stabilise lipid rafts (LR), which are regions of cell membranes with a distinct lipid composition and which appear to act as platforms, called “ceramide-enriched membrane platforms” [13,14], to localize proteins involved in intracellular signalling. The contribution of lipid rafts to the efficient activation of the NADPH oxidase has been investigated in neutrophils in several studies [15–18]. It was also hypothesised that lipid raft platforms with aggregated NOX-4 subunits form a number of lipid raft associated NOX-4 complexes, which results in increased production of O_2_^. -^ by endothelial cells [14].

The association between low antioxidant defence and increased biomarkers of oxidative damage in periodontitis plasma has been investigated in several studies [3]. Whilst biomarker levels of oxidative damage such as 8-hydroxydeoxyguanosine (8-OHdG), lipid peroxidation products [19], and protein carbonyl [20] levels are found to be increased in patients with periodontitis, serum enzymatic antioxidants such as superoxide dismutase (SOD), catalase (CAT), glutathione peroxide (GSHPx), and non-enzymatic antioxidants (GSH, vitamins E and C) are found to be significantly lower [19] than healthy control subjects. The extracellular redox environment has been suggested to impact on intracellular redox state and cellular metabolism [7,21]. We have previously shown that even after non-surgical therapy, extracellular GSH levels remain low in gingival crevicular fluid in chronic periodontitis patients than disease free controls [7]. The exogenous ROS burden creates an ‘oxidatively stressed’ environment mainly by generating intracellular H_2_O_2_, which acts as a second messenger in cell signalling pathways. H_2_O_2_ reacts with freely accessible protein thiolate anions to form cys-sulfenic acids or is further oxidised to disulfide bonds. Most of these reactions are reversible in the presence of cellular thiol/disulfide systems; GSH/GSSG, thioredoxin/ thioredoxin reductase, and cysteine/cystine [22]. However, in the presence of excessive oxidation, cysteine and GSH depletion can result, which eventually overwhelms endogenous oxidation-reduction (redox) reactions. Intracellular redox status is very important in protein function and to maintain cellular homeostasis, however, whether the intracellular redox state is altered in periodontitis is unknown.

Nuclear factor (erythroid-derived 2)-related factor 2 (Nrf2) is a helix–loop–helix basic leucine zipper transcription factor retained in the cytoplasm that is subject to redox regulation. Nrf2 is expressed by nearly all cell types, where its function is suppressed and sequestered in the cytosol by the Kelch-like Erythroid-cell-derived protein with CNC homology (ECH)-Associated Protein 1 (Keap1). Two reduced cysteine residues (cys273 and cys288) in Keap1 are required for ubiqutination of Nrf2 [23,24]. Keap1-Nrf2 interaction prevents Nrf2 nuclear translocation and so prevents activation of target genes through binding to the antioxidant response element (ARE). Upon challenge by oxidants Nrf2 does not undergo ubiquitination and is released from Keap1 as a result of the modification of cysteine residues in Keap1 and/or phosphorylation of Nrf2. Activated Nrf2 translocates to the nucleus, where it binds to promoters and upregulates Nrf2-ARE target genes such as heme oxygenase-1 (HMOX1), glutamate cysteine ligase catalytic (GCLC), and modifier (GCLM) subunits, and NADPH-quinone oxidoreductase (NQO1). Cellular and extracellular glutathione concentrations are therefore under redox control via Nrf2; and the loss of intracellular glutathione increases cellular peroxides, promotes release of Nrf2 from Keap1 following thiol oxidation, triggers increased de novo glutathione synthesis via the rate limiting enzyme γGCS and restoration of the cellular redox state. Further regulation of the Nrf2 pathway is achieved via removal of oxidised Keap 1, which has recently been reported to undergo autophagic turnover in liver cells [25].

Sulforaphane (SFN) is a natural product found in cruciferous vegetables. Although the exact mechanism is not fully understood, SFN is known to induce Nrf2 dependent antioxidant gene expression by binding to cys151 on Keap-1 [24]. SFN also acts as an inhibitor of histone deacetylase (HDAC); which is involved in altered histone acetylation status and increased p21^Cip1/Waf1^ expression in human embryonic kidney cells [26]. The protective effects of SFN against oxidative damage have been studied in various *in vitro* [27] and *in vivo* [28] models and recently, the role of Nrf2 activity in modulating innate immune responses has been investigated in sepsis [29].

To date, neutrophil redox state, Nrf2 expression and activity have not been studied in subjects with periodontitis. Here we test the hypothesis that chronic inflammatory periodontitis is associated with impaired Nrf2 activation and function, insufficient glutathione synthesis and depletion of the glutathione pools, which in turn mediates a hyperactive and hyper-reactive neutrophil phenotype. We have also investigated a protective effect of SFN in reducing periodontitis patient neutrophil hyperactivity ex vivo.

## Materials and Methods

### Materials

Anti-Nrf2 polyclonal antibody was purchased from Invitrogen (Paisley, UK). All other reagents were obtained from Sigma Chemical Company (Poole, UK) unless otherwise stated.

### Volunteers

Consenting volunteers were recruited from chronic periodontitis patients (n=15; eight males and seven females; age range 43-57 years) attending Birmingham Dental Hospital. Chronic periodontitis was defined as previously described [30]. Age- and sex-matched periodontally healthy consenting control subjects (n=15; eight males and seven females; age range 40-60) were recruited from staff of the Birmingham Dental Hospital. All volunteers were systemically healthy, non-smokers, not pregnant and did not use recreational drugs at the time of sample collection. Ethical approval for the study was granted by South Birmingham Local Research Ethics Committee (South Birmingham LREC 05Q/2707/252; Am01/1) and donors gave their informed written consent after the risks and benefits of partaking in the study were explained.

### Collection and isolation of peripheral blood neutrophils

Venous blood was collected into lithium heparin (17 IU/ml) Vacutainer™ (Greiner Bio One Ltd.) tubes. Neutrophils were isolated as described by Matthews et al [3] using Percoll^®^ density centrifugation (Invitrogen, Paisley, UK). Isolated cell viability was determined immediately before analysis by trypan blue exclusion and was typically >98%. Primary neutrophils were cultured in RPMI1640 media containing 10% foetal bovine serum and 200U/ml penicillin and streptomycin in the presence or absence of 5µM SFN for 16 hours. Viability was determined by trypan blue exclusion.

### Cell culture

Human promyelocytic (HL60) cells from ATCC were maintained in RPMI 1640 media containing 10% foetal bovine serum and 200U/ml penicillin and streptomycin at 37^0^C in a humidified atmosphere of 5% CO_2_ and 95% air. HL60 cells were differentiated into a neutrophil-like cell line (dHL60) by maintaining cells in media containing 1% DMSO for 5 days.

### Chemiluminescence assay for extracellular superoxide anion radical production

To determine the respiratory burst activity of resting and activated neutrophils, lucigenin-dependent chemiluminescence was monitored over one hour as previously described [4]. Briefly, PBS-washed primary neutrophils (5 × 10^5^ cells) in 100 µL of 1% BSA-PBS buffer were incubated with 100 µM lucigenin in white microplate wells previously blocked with 1% BSA overnight. After equilibration to 37°C for 30 min, light emission in relative light units (RLUs) was recorded in order to study baseline/resting superoxide anion radical release for 30 min. This was followed by the addition of fMLP (1 µM), 25µl of opsonised *S. aureus* (300 bacteria/neutrophil), *F. nucleatum* suspension (heat killed non-viable bacteria x100/neutrophil), 10nM phorbol 12-myristate 13-acetate (PMA) or PBS (unstimulated control) to stimulate the respiratory burst, and further measurements were recorded for another 30 min. All samples were analysed in triplicate, with paired patient and control samples analysed at the same time, and under the same conditions. Mean maximum RLUs were plotted for each experiment.

### Measurement of intracellular GSH and GSSG levels

After 16 hours incubation, SFN-treated cells and non-treated control neutrophils (5×10^5^ cells) were pelleted, washed twice with PBS and the pellet was air dried for 5min. Sulfosalicylic acid (SSA; 3.33µl of 100% made up in distilled water) was then added to the cell pellet, vortexed and immediately centrifuged at 6600 ×g for 1.5 min. Stock buffer (96.6µl of 125mM sodium phosphate, 6.3mM disodium EDTA, pH 7.5) was then added to each tube, vortexed and re-centrifuged as above. Supernatants were collected into fresh tubes and GSH and GSSG levels were assessed by the GSR-DTNB recycling assay as described in Gherghel D, et al. [31], on the same day or samples were immediately stored at -80^0^C for analysis within one month. Protein concentration was measured by bicinchoninic assay (BCA assay).

### Measurement of acid sphingomyelinase (ASMase) activity

ASMase activity in dHL60 cells was measured as described previously with some modifications [32]. dHL60 cells (1×10^7^) were centrifuged and washed with ice-cold PBS to remove media. The cell pellet was resuspended in 1ml of a lysis buffer (25mM Tris–HCl buffer (pH 5), 2mM EDTA, 2mM EGTA, 1mM phenylmethylsulfonyl fluoride, 20µg/ml E-64). Cell extracts were homogenised by passing through a 25G needle 5 times and used as the enzyme source.

The assay mixture contained the following components in a total volume of 200µl: 15mM HADPC (2-N-hexadecanoylamino-4-nitrophenylphosphoryl choline; Calbiochem, UK), 100mM Tris–HCl buffer (pH 5), 10mM MgCl_2_, and 10µl of the enzyme source. Incubation followed at 37^0^C for 60 min; the enzyme reaction was terminated by adding 400µl 100mM glycine buffer (pH 10.5) and 700µl ethanol. The suspension was vortexed and centrifuged at 2000 × *g* for 10 min. The absorbance of the supernatant solution was measured spectrophotometrically at 410 nm.

### Isolation of lipid raft microdomains by gradient centrifugation

dHL60 cells (1×10^7^ cells) were lysed in 1 ml MNE buffer (150mM NaCl, 2mM EDTA, 25mM MES, with 1% protease inhibitor cocktail, pH6.5) containing 1% Triton X-100 on ice for 30min. Cell extracts were homogenized by 5 passages through a 21-gauge needle. Lysates were obtained by centrifuging at 14,000*g*, 4°C for 5 min to remove the nuclei and insoluble materials. The cell lysates (1ml) were mixed 1:1 with 85% sucrose solution, layered in the bottom of the centrifuge tube (Ultra clear™ Beckman centrifuge tubes) and overlaid sequentially with 6ml 30% and 3.5ml 5% sucrose solution to make a non-continuous sucrose gradient. Samples were centrifuged at 20 000g for 16 hours at 4°C using a SW41Ti rotor (Beckman). Nine 1ml fractions were collected from the top and proteins in each fraction were precipitated with 5% trichloroacetic acid for 30 minutes on ice. Proteins in lipid raft (LR) fractions were isolated by centrifugation at 13 000 x g, 4°C, for 15 minutes. The protein pellet was carefully washed with cold acetone twice, air dried, and then resuspended in modified Laemmli buffer (4M urea, 0.2% ABF-14, 20% DMSO, 4% SDS, 20% glycerol, 10% 2-mercaptoethanol, 0.004% bromophenol blue and 0.125M Tris-HCl, pH 6.8). Samples were heated 5 min at 95^0^C before storage at -20^0^C for later analysis by western blot.

### Western blot analysis of proteins in lipid rafts

For immunodetection of LR-associated proteins, 15µl of each fraction in modified Laemmli buffer were subjected to 10% SDS-PAGE, transferred onto a PVDF membrane, and blocked with 3% BSA. The membrane was probed with primary monoclonal antibodies anti-flotillin-1 (1:1000, BD Biosciences), for 2 hours at room temperature followed by extensive washing then incubation with horseradish peroxidase–labeled anti-rabbit IgG (1:5000) for 2 hours. The immunoreactive bands were detected by enhanced chemiluminescence methods (GE HealthCare).

### Preparation of nuclear and cytosolic extracts and Nrf2 activity assay

Nuclear and cytosolic extractions (3×10^5^ cells) were prepared using the Active Motif Nuclear extraction kit (Active Motif, Carlsbad, Calif) according to the manufacturer’s instructions. Samples were immediately stored at -80^0^C. Protein concentrations were measured spectrophotometrically (NanoDrop). Nrf2 nuclear binding was assessed by using an ELISA based Nrf2 TransAM transactivation kit (Active Motif, Carlsbad, Calif) according to manufacturer’s instructions. Briefly, five µg of nuclear protein was incubated in 96-well plates pre-coated with ARE consensus oligonucleotides, and the active-Nrf2 that bound to the oligonucleotide was detected HRP-conjugated secondary antibody. The absorbance was read using a plate reader at 450nm, and absorbance was expressed as the direct activity of Nrf2.

### RNA extraction and quantitative polymerase chain reaction (qPCR)

Nrf2-regulated antioxidative gene expression was measured in neutrophils by quantitative PCR. Total RNA was extracted from the cells (1x10^6^) using TriZol reagent (Invitrogen, Carlsbad, CA) according to the manufacturer’s instructions. RNA was treated with DNase (Qiagen) for 30 minutes at room temperature. RNA was subsequently purified using the RNAeasy Kit (Qiagen). RNA quantification was performed using the Nanodrop 1000 (Thermofisher). 1 µg of total RNA was reverse transcribed using Precision nanoscriptTM reverse transcriptase (Primerdesign, Southampton UK) and oligo dT primers (PrimerDesign, Southampton, UK). cDNAs were amplified in a standard 40-cycle SYBR® green real-time PCR reaction using optimised sequence specific pre-validated primers supplied by PrimerDesign Ltd (Southampton UK), according to the manufacturer’s instructions. cDNA was used for quantitative PCR analyses of selected genes heme oxygenase-1 (HMOX1), glutamate cysteine ligase catalytic (GCLC), and modifier subunits (GCLM), and NADPH-quinone oxidoreductase (NQO1) using primers commercially available from PrimerDesign Ltd (Southampton UK). β-Actin was used for normalization. Fold changes in gene expression using the comparative CT method and statistical analysis were determined using the freely available Relative Expression Software Tool (REST 2009, www.qiagen.com).

### Preparation of whole cell extracts for Nrf2 and Keap 1 western blot

Neutrophils were pelleted, washed twice with ice-cold PBS and resuspended in 100µl of ice-cold lysis buffer [50 mM Tris-HCl (pH 6.8), 10% (vol/vol) glycerol, and 2% (wt/vol) SDS]. After 10 min on ice, cells were sheared by passing five times through a G21 needle and syringe to reduce DNA viscosity. Samples were centrifuged for collection of the supernatants containing total proteins. Protein concentration was measured using BCA assay. Laemmli sample buffer (100µl) was added, vortexed and stored at -20^0^C. Western blot analysis for Nrf2 was performed as described previously [33] using 20µg of isolated soluble proteins. To detect Keap-1, membranes were blocked with 3% BSA in PBS-T (0.05% Tween 20 in PBS) for 2hrs, treated with primary antibodies overnight followed by HRP-conjugated secondary antibody for 2hrs. Protein signals were developed with ECL Plus reagents (Amersham). For quantification, densitometric integration of the bands of interest was undertaken using a GS800 scanner with Quantity One (version 1.34r; Biorad).

### Data analysis

Data were analysed using Graphpad Prism software (version 5). Unless specified all data are presented as the mean±SEM of at least three independent experiments, performed in triplicate. Statistical analysis was performed using analysis of variance followed by Tukey’s multiple comparison test.

## Results

### Sulforaphane increases intracellular GSH/GSSG ratio and decreases the PMA stimulated respiratory burst in dHL60 cells

**Figure 1:**
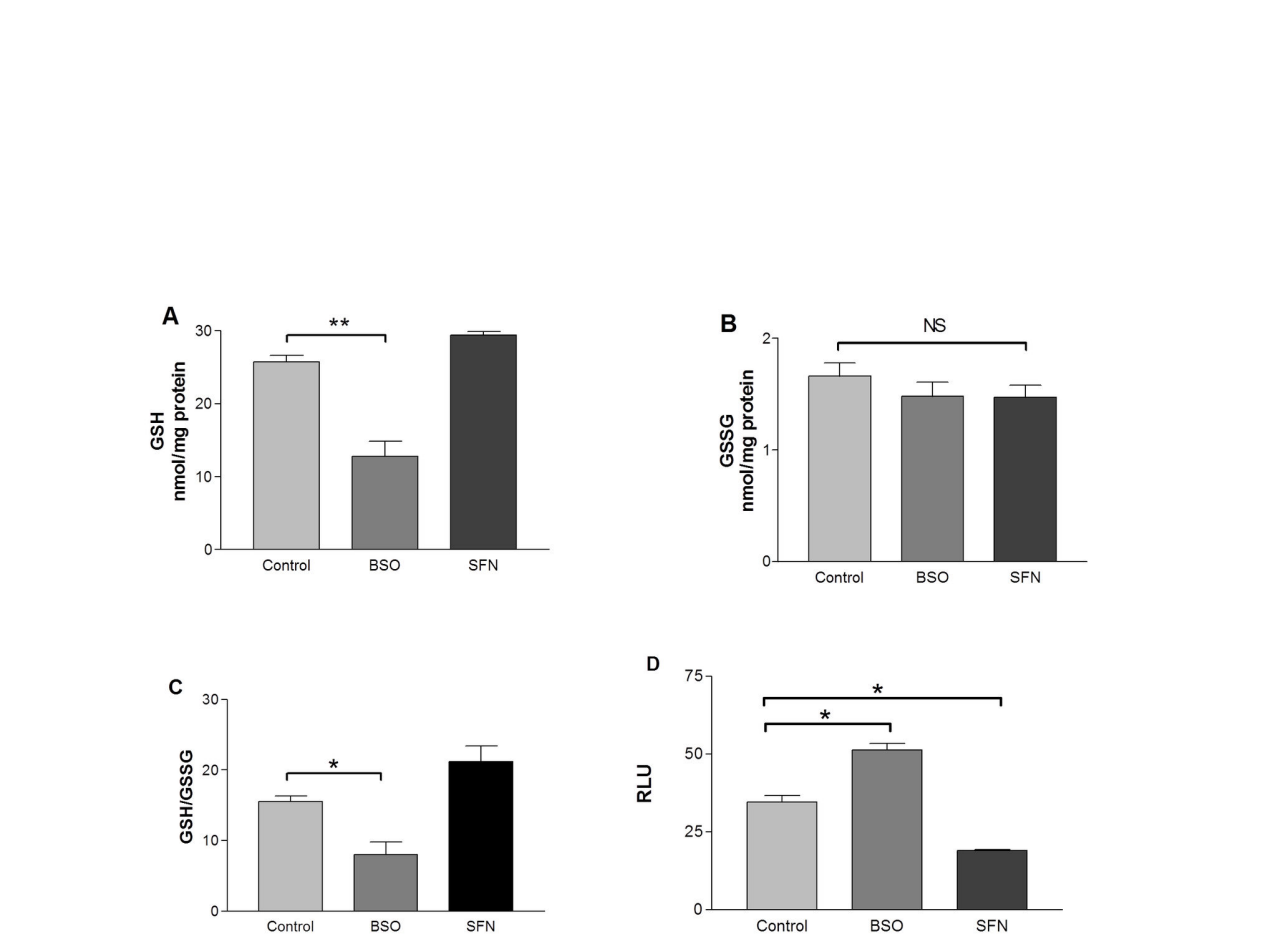
Effects of BSO and SFN pre-incubation on dHL60 cell GSH/GSSG ratio and respiratory burst. dHL60 cells were treated with 10µM BSO or 5µM SFN for 16 hours. GSH (A) and GSSG (B) concentrations were measured by the DTNB recycling assay in order to determine the GSH/GSSG ratio (C). Mean peak lucigenin chemiluminescence generated by dHL60 cells was plotted (RLU ± standard error of the mean) (D). Data represent three independent experiments of three replicates. Significant differences were calculated with one way ANOVA followed by Tukey’s multiple comparison test, where *P<0.05.

To study the change in intracellular redox environment on dHL60 cell response to stimulus and the effect of SFN, intracellular GSH (Figure 1A) and GSSG (Figure 1B) levels were measured pre- and post-SFN treatment. The glutathione synthesis inhibitor; BSO (10µM for 16 hours) significantly decreased intracellular GSH concentration in dHL60 cells (12.8±2 nmol/mg protein; P<0.01) compared to untreated control cells (25.8±0.9 nmol/mg protein). SFN (5µM for 16 hours) increased intracellular GSH levels to 29.4±0.5 nmol/mg protein. The change in intracellular GSSG levels was not significant between treatments. As a result, the dHL60 cell GSH/GSSG ratio (Figure 1C) was significantly decreased with BSO treatment and GSH/GSSG appeared higher following SFN treatment. Cell viability was not affected by any treatment (Figure S1 in File S1).

In order to investigate the dHL60 cell respiratory burst under GSH depletion with BSO, cells were stimulated with 10nM PMA (Figure 1D). PMA enhanced the lucigenin-sensitive respiratory burst in BSO pre-treated dHL60 cells (51.3± 2.1RLU; P<0.05) compared to controls (34.6 ± 2.1RLU). A significant decrease in the respiratory burst (18.9± 0.4RLU; P<0.05) response to PMA was observed with SFN treated dHL60 cells. SFN treatment did not change phagocytic ability of dHL60 cells as measured by flow cytometry suggesting that the effect of SFN on the respiratory burst is not attributed to a generally decreased functional activity (Figure S2 in File S1).

### dHL60 cell ASMase activity and lipid raft rearrangement is increased by GSH: GSSG depletion

**Figure 2:**
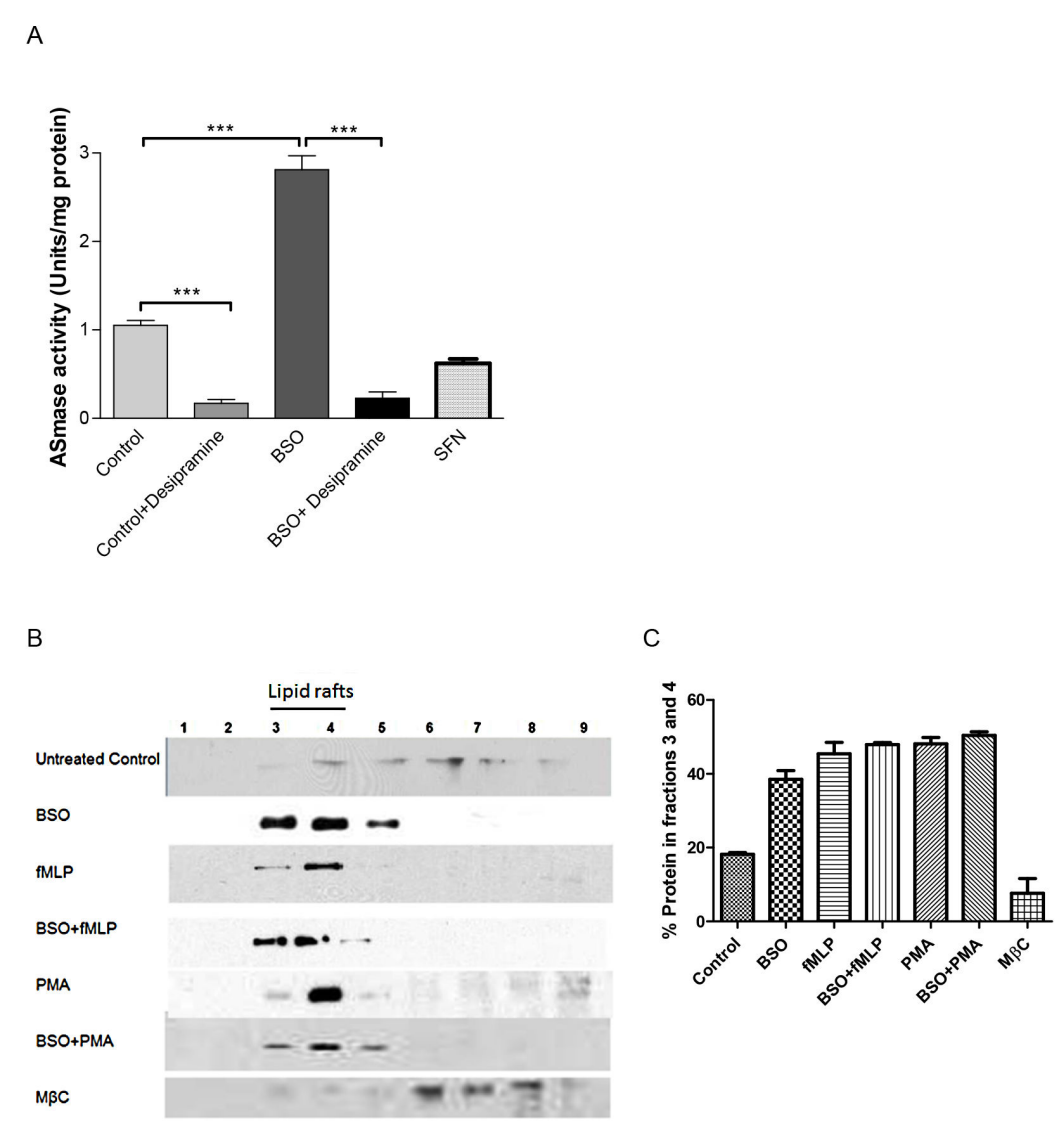
Effects of oxidative stress on ASMase activity and LR rearrangement. (A) BSO (10µM) pre-treated and untreated dHL60 cells were treated with or without desipramine (10 µM, 1 hour) and 5µM SFN. Cells (1×10^7^) were collected on ice-cold PBS, pelleted and lysed before analysing ASMase activity. (B) LRs from dHL60 cells were extracted with MNE buffer containing 1% triton X-100 and applied to discontinuous sucrose gradients, as described in Methods. Proteins were extracted and immunoblotted using anti-flotillin-1 as the raft protein detecting antibody. (C) Quantification of flotillin in lipid raft fractions 3 and 4 compared to total expression.

The change of intracellular redox environment following BSO treatment may affect the activity of redox sensitive proteins such as ASMase (Figure 2A). dHL60 cells treated with 10µM BSO for 16 hours increased ASMase activity by 2.5 fold compared to the control cells (P < 0.001). Desipramine, an inhibitor for ASMase significantly decreased ASMase activity with in both BSO untreated control (10 fold) and BSO treated cells (5 fold; *P* < 0.05). SFN, an inducer for Nrf2 nuclear translocation significantly decreased ASMase activity by 2 fold compared to untreated control cells (P<0.05). Lipid rafts (LR) are re-arranged in redox stressed and stimulated dHL60 cells (Figure 2B). Compared to untreated dHL60 cells, fMLP and PMA stimulated cells are rich in the LR marker protein flotillin-1 in fractions 3-4. BSO treated dHL60 cells show further up-regulation of flotillin-1 into LR fractions. The cholesterol-depleting agent 10mM methyl-β-cyclodextrin (MβC) for 1 hour induced redistribution of lipid raft marker protein, flotillin into non-raft fractions.

### SFN increases intracellular glutathione in primary neutrophils

**Figure 3:**
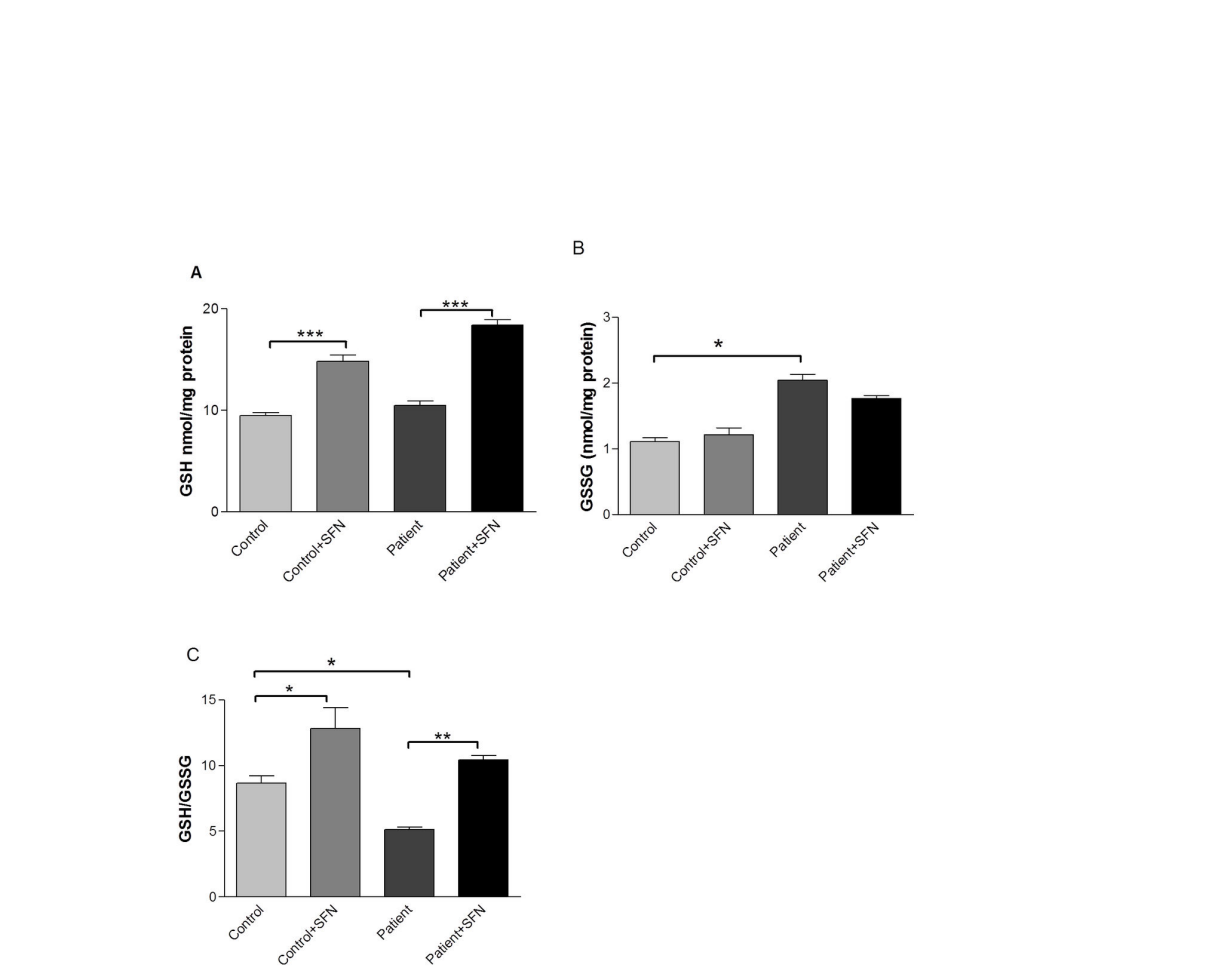
SFN pre-incubation increases the GSH/GSSG ratio in primary neutrophils. GSH (A) and GGSG (B) concentrations, along with the GSH/GSSG ratio (C) were measured using the DTNB recycling assay and expressed relative to protein content. Significant differences were calculated with Tukey’s multiple-comparison test where*P<0.05, and ***P<0.001.

To determine whether *in vitro* cell culture studies were reproducible in periodontitis patients, who have previously been reported to have lower extracellular GSH concentrations [5], blood samples were obtained and primary neutrophils were isolated from 12 periodontitis patients and age/gender matched healthy volunteers. The redox state of neutrophils was determined by analysing the intracellular GSH/GSSG ratio (Figure 3). Patient neutrophils were significantly higher in GSSG concentration (2.04±0.1nmol/mg protein) compared to controls (1.16± 0.07nmol/mg protein). Pre-incubation with SFN for 16 hours significantly increased intracellular GSH concentrations in both control (14.83±0.6 nmol/mg protein) and patient (19.62±0.5 nmol/mg protein) groups. The levels of GSSG in SFN-treated control (1.2±0.11 nmol/mg protein) and patient (1.76±0.04 nmol/mg protein) cells were unaffected. The intracellular GSH/GSSG ratio was significantly increased after 16 hours of SFN treatment in both patients (P<0.01) and control subjects (P<0.05).

### SFN reduces neutrophil respiratory burst

**Figure 4:**
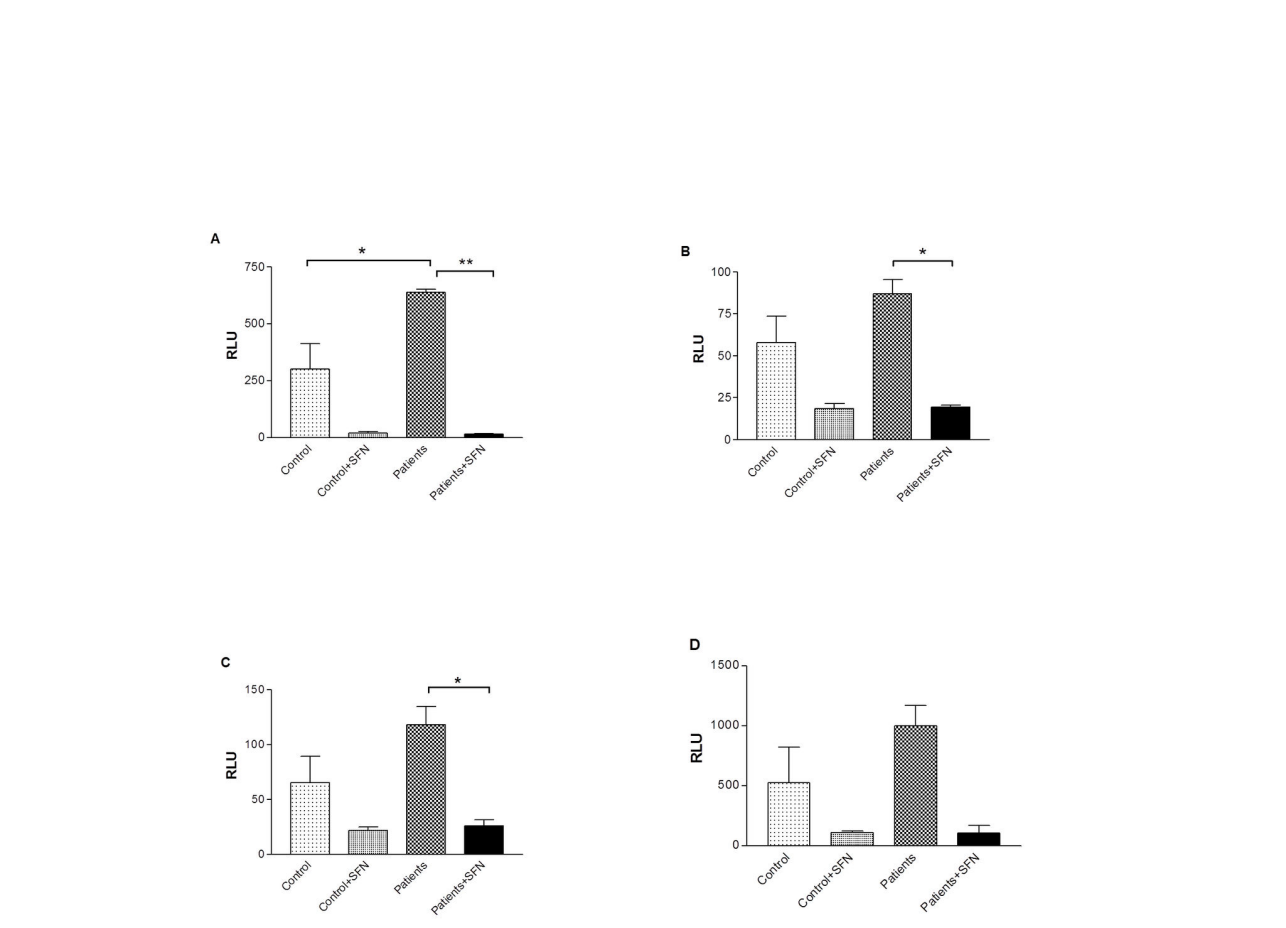
SFN pre-incubation decreases neutrophil respiratory burst. Peak relative light unit (RLU) values for neutrophils stimulated with (A) 1µM fMLP, (B) opsonised *S. aureus* (300 bacteria/neutrophil) (C) *F. nucleatum* bacteria x100/neutrophil and (D) 10nM PMA in the presence or absence of SFN treatment were plotted and significant differences were calculated using Tukey’s multiple-comparison test, where, * p<0.05 and **p<0.01.

In order to investigate the effect of SFN on the neutrophil respiratory burst, primary neutrophils were pre-treated with SFN or vehicle for 16 hours. Neutrophils were stimulated with fMLP, opsonised *S. aureus*, heat-killed *F. nucleatum* (60^°^C) or PMA (Figure 4 A–D respectively) and lucigenin-dependent chemiluminescence was measured. Periodontitis patients’ neutrophils showed significantly greater hyper-reactivity towards all four stimulations compared to neutrophils from healthy subjects (p<0.05). SFN treatments did not significantly change neutrophil cell viability measured at 16 hours by trypan blue (Figure S3 in File S1). Both control and patient neutrophils showed decreased lucigenin-dependent chemiluminescence in response to all stimuli after SFN treatment. While extracellular superoxide production measured by lucigenin-dependent chemiluminescence is inhibitable by the addition of superoxide dismutase at the peak of the respiratory burst, addition of extracellular N-acetyl-cysteine (NAC) or SFN on maximally activated neutrophils did not quench the signal (Figure S4 in File S1).

### SFN increases primary neutrophil Nrf2 levels

**Figure 5:**
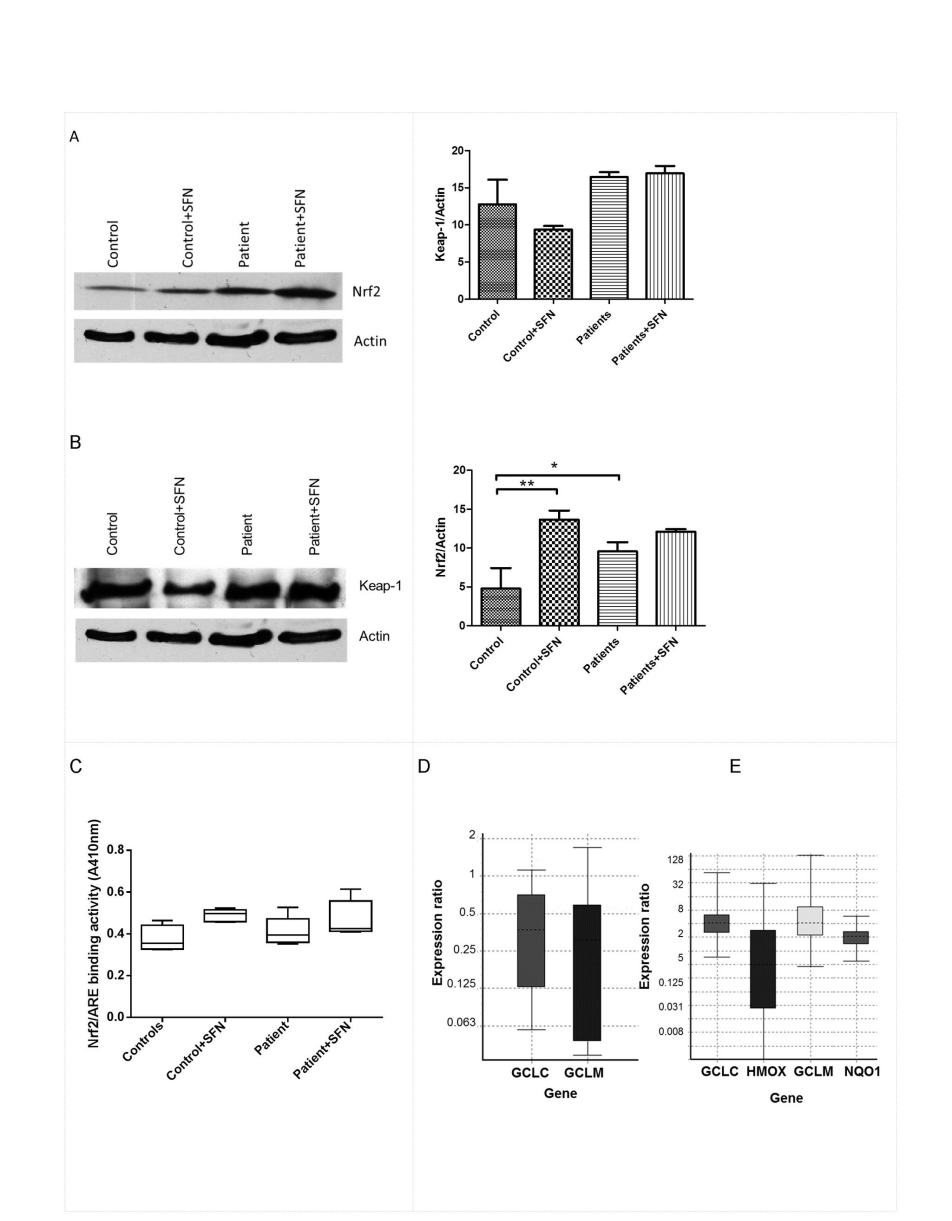
Effect of SFN on Nrf2 protein expression and activity in primary neutrophils. (A) Western blotting (left) and quantification (right) of total Nrf2 expression in the presence or absence of SFN treatment. (B) Western blotting (left) and quantification (right) of expression of Keap-1, Values are shown normalised to β-actin. (C) Analysis of Nrf2-DNA binding activity by trans-AM nuclear activity assay. A representative experiment of the three is shown. *** P< 0.01. (D) qPCR analysis of GCLC and GCLM gene expression levels of neutrophil mRNA from patients compared to control. (E) qPCR analysis of GCLC, HMOX1, GCLM and NQO1 gene expression levels in neutrophils treated with 5 µM SFN for 16 hours, values normalised to actin.

Low doses of SFN exert indirect antioxidant properties via Nrf-2 mediated gene expression. To investigate whether SFN prevents the neutrophil respiratory burst by up-regulation of Nrf2, cell lysates were examined for Nrf2 expression by immunoblotting. Total Nrf2 expression was examined by western blotting after treatment with different doses (0, 1, 2, 5, 10 or 15µM) of SFN for 2, 4, 8, 16 and 24hrs. Exposure of neutrophils to increasing concentrations of SFN led to an increase in total Nrf2 level (Figure S5A in File S1). Incubation with 5, 10 and 15µM SFN resulted in an increase in Nrf2 protein level by 68%, 70% and 77% over control cells, respectively. In time course experiments a significant nuclear translocation of Nrf2 was observed after 4hrs treatment with 5µM SFN and this was further increased at 8 and 16 hrs (Figure S5B in File S1). Nuclear translocation of Nrf2 at 24hrs was not significantly different to 16hrs. Keap-1 protein level was reduced at 4 hrs but restored at 16 hrs (Figure S5B in File S1). Figure 5A shows that SFN increases Nrf2 protein levels in neutrophil cell lysates from control subjects. In the absence of SFN, patient neutrophils show increased Nrf2 expression compared to healthy control cells. Keap-1 protein levels were not different between control and patient neutrophils in the absence of SFN but were depleted in control subjects only after SFN treatment for 16hrs (Figure 5B). Nrf2-ARE binding efficacy (Figure 5C) was not significantly different between control (0.38±0.03 arbitrary units; AU) and patients (0.41±0.03AU). SFN treatments increased DNA binding activity in both control (0.49±0.01AU) and patients (0.47±0.04AU) nuclear fractions. The transcription factor Nrf2 regulates the gene expression of a number of phase II genes such as HMOX1, GCLC, and GCLM, and NQO1, which increases cellular glutathione (GSH) synthesis. Figure 5D shows that GCLC and GCLM relative mRNA expression levels were significantly down regulated in the patient group by a mean factor of 0.305 and 0.215 respectively (P<0.05). SFN treatments for 16 hours significantly increased relative mRNA expression in both groups for GCLC, GCLM and NQO1 (Figure 5E) but not HMOX1 where the lack of response was in patient neutrophils but not in normal subjects.

## Discussion

**Figure 6:**
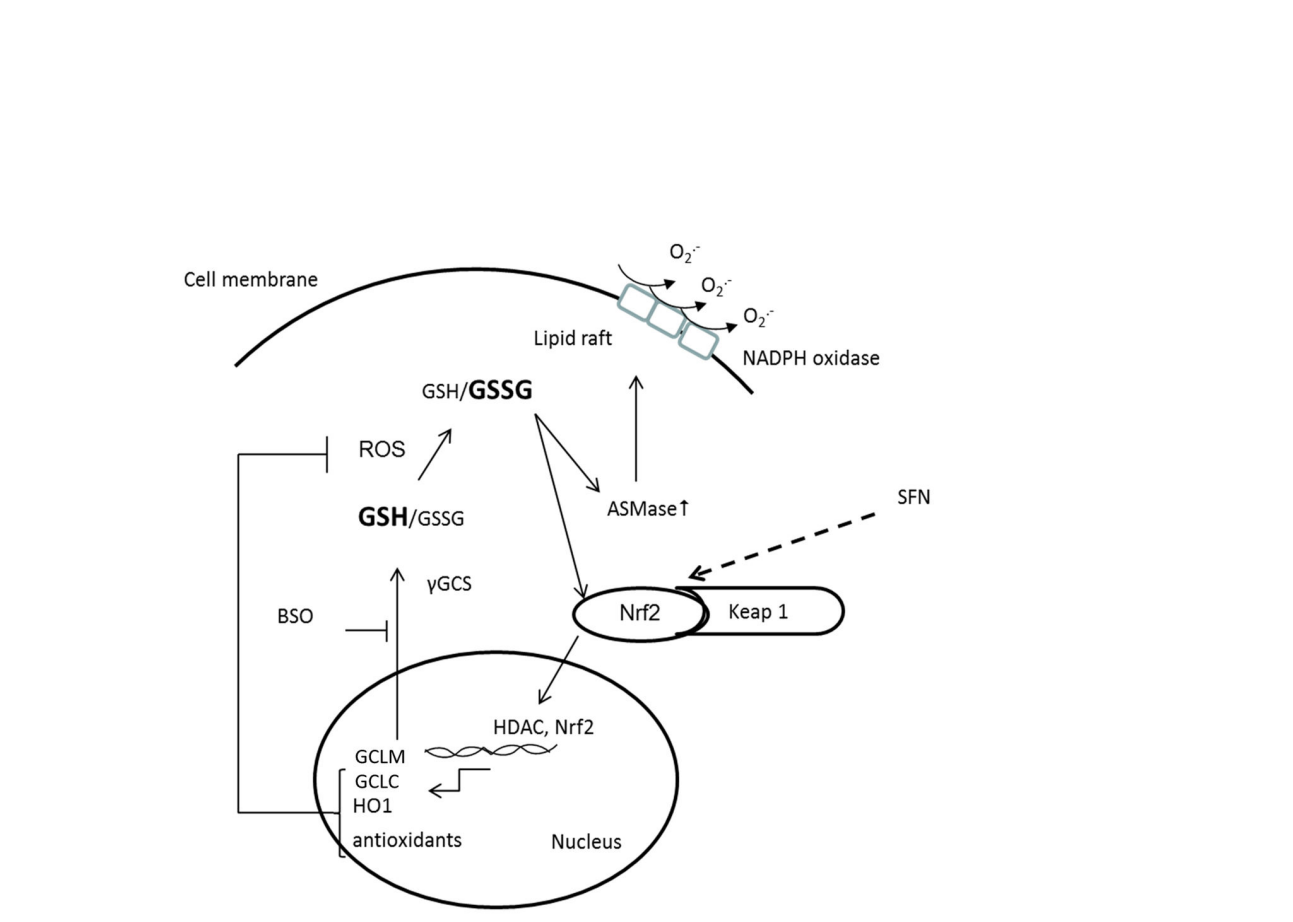
A possible mechanism for the up-regulation of NADPH oxidase activity under redox stress. The pathway leading to the formation of GSH by the action of γ-glutamylcysteine synthetase (γGCS) is blocked by buthionine sulfoximine (BSO), inducing artificial stress condition in dHL60 cells. Decreased cellular GSH/GSSG ratio may activate redox sensitive enzymes such as ASMases and nuclear translocation of Nrf2. ASMases may favour lipid raft formation and thereby clustering active NADPH oxidase complexes in the outer membrane, subsequently liberating extracellular O_2_^.-^.

The present study provides evidence to show that peripheral blood neutrophils from patients with chronic periodontitis have more oxidised (GSSG) relative to reduced glutathione (GSH) compared to neutrophils from healthy control subjects. Normally, the Keap1-Nrf2-ARE signalling pathway elicits an adaptive response for cell survival under oxidative stress but this did not appear to operate effectively in periodontitis patients. To our knowledge this is the first study to reveal disruption of the Nrf2 pathway as a mechanism underlying redox disturbances and neutrophil hyperactivity/hyper-reactivity in periodontitis. The results also demonstrate that SFN can restore the intracellular redox state measured by GSH/GSSG in neutrophils from controls and periodontitis patients ex vivo, although this may be independent of Nrf2 activity. SFN increased the expression of GCLC and GCLM mRNA and GSH/GSSG ratio in both healthy primary neutrophils and periodontitis patients mimicking the effect of SFN on GSH/GSSG seen in differentiated HL60 cells. Subsequently, both the primary neutrophil and dHL60-stimulated respiratory burst was significantly decreased with SFN treatments. The potential mechanism by which SFN exerts such protection is represented in schematic Figure 6.

Healthy cells generally maintain a reducing intracellular environment, relative to extracellular fluid compartments. A major factor influencing the redox status of the cytosol is the concentration of reduced glutathione [34]. It has been estimated that cultured cells maintain a cytosolic GSH/GSSG redox potential of -260mV to -200mV compared with the more oxidised redox potential found in plasma, -140mV [35]. Pathophysiological conditions such as inflammation and ageing [36] drive the cytosolic GSH/GSSG-coupled redox potential to be more positive (oxidising). Experimentally, BSO has been widely used to induce GSH depletion (hence increase redox potential +30mV at 100µM BSO for 24hrs in HeLa cells [37]) due to its inhibitory effects on γGCS production, the rate limiting enzyme in GSH synthesis [38]. The use of differentiated HL60 cells as a valid model system to resemble the functional properties of primary neutrophils has been reported in several studies [39,40]. Differentiated HL60 cells exposed to BSO for 16 hours showed a significantly depleted cellular glutathione pool. However, the GSSG level was generally maintained in dHL60 cells after treating with BSO for 16 hours. This created a decrease in cellular GSH/GSSG ratio indicative of a more oxidised cellular environment. Compared with healthy controls, patients with periodontitis had higher GSSG concentrations whereas GSH levels were not significantly different. As a result, patient neutrophils exhibited a GSH/GSSG redox ratio consistent with a more oxidised state. Cells generally maintain a reduced intracellular environment by recycling GSH using the enzyme glutathione reductase, and by exporting excess GSSG out of the cell. When these cycles are overwhelmed, other cellular proteins may be irreversibly oxidised. It has been shown that antioxidant treatments will enhance the neutrophil redox state and the GSH/GSSG ratio [41].

Nrf-2 is a transcription factor which regulates the basal and inducible expression of a wide array of antioxidant genes. Although the redox disturbances seen in periodontitis neutrophils were accompanied by an expected upregulation of Nrf2 expression, this did not translate to an increase in Nrf2-ARE binding which instead was at the same level as control subject neutrophils. Furthermore, Nrf2 targeted gene expression was significantly down regulated for GCLC and GCLM between control and patients neutrophils in the absence of any ex vivo activating stimulus. However, with SFN treatment ex vivo, neutrophil HMOX1, GCLC and GCLM expression between control and patients was increased several fold compared to non-treated control cells but NQO1 was only increased in control subject neutrophils. Differential regulation of HMOX1 and NQO1 is reported in ethanol induced murine models of inflammation illustrating the complexity of interactions between different transcription factors and such complexity of transcription factor interactions may also underpin the apparent failure to adapt to increasing GSSG in patient neutrophils despite elevated Nrf2 levels [42]. Recent studies have described the importance of Keap1 turnover after oxidation via autophagic degradation paralleled by upregulation of de novo transcription via redox sensitive transcription factors AP2 and Sp1 [25]. Of note, turnover in Keap1 was evident in control subjects but not patients and this may indicate greater Keap 1 activity in periodontitis neutrophils which in turn may afford greater inhibition on Nrf2. Together, this suggests that the Nrf2 pathway in neutrophils is disrupted and cannot facilitate adaptation to oxidative stress in chronic periodontitis. Therefore the high expression of Nrf2 in unstimulated primary neutrophils in patients does not appear to recover the cellular redox balance in response to GSSG elevation. Alterations of the Nrf2 pathway have been previously described in other diseases such as chronic pulmonary disease [43], diabetic nephropathy [44] and neurodegenerative diseases [45].

This study has not investigated the specific mechanism underlying Nrf2 dysfunction in periodontitis. It is possible that in addition to potential over activation of Keap 1 or lack of Keap1 turnover, Nrf2 in neutrophils is post-translationally modified in periodontitis patients, which might interfere with its ability to undergo nuclear trafficking. Post translational modifications of Nrf2 including acetylation [46], phosphorylation [47] and alterations in Keap1-dependent ubiquitination [33] are reported in several studies. With the addition of SFN, the redox balance of patient neutrophils was restored independently of Nfr2-ARE binding, and associated with decreased hyperactivity/hyper-reactivity. SFN is able to react with the thiol groups of Keap 1 and form thionacyl adducts promoting Nrf2 dissociation from Keap1 and subsequent activation of ARE driven gene expression and this may explain the increased GSH levels measured in control cells following SFN treatment. However, they do not explain the restoration of GSH in periodontitis neutrophils, implicating an Nrf2 independent pathway for SFN protection.

We have previously demonstrated lower GSH concentrations in periodontitis patients relative to healthy controls within the gingival crevicular fluid that bathes the periodontal tissues [48]. Moreover, mechanical anti-infective therapy, whilst successful at reducing clinical inflammation, failed to restore GSH concentrations to control patient levels, implying a dysfunctional GSH synthesis pathway in periodontitis [7]. The failure to adapt to sustained oxidative stress via Nrf2 antioxidant mechanisms may create an intracellular redox shift. Redox sensitive proteins such as ASMase could become activated in such environments. Evidence suggests that a more oxidised intracellular redox state can activate ASMases [49]. Ceramide-rich membranes form stable cellular platforms, where more NADPH oxidase complexes cluster together, as reviewed in Li et al. [14],. Previous studies have shown that NADPH oxidase subunit redistribution into lipid rafts could be initiated by different stimuli [15,50,51]. Upregulation of the respiratory burst in response to a PMA stimulus in GSH-depleted dHL60 cells suggests that this environment favours NADPH oxidase activity. Therefore, the more oxidising intracellular redox state may affect NADPH oxidase membrane assembly by altering lipid raft structure and promote hyperactivity.

In summary, we have demonstrated for the first time that significant redox disturbances exist in neutrophils of patients with periodontitis and are associated with dysregulation of the anti-inflammatory transcription factor Nrf2. Activation of the redox sensitive protein ASMase, to promote lipid raft formation and thereby assembly of the NADPH oxidase enzyme was also mediated by a disruption in redox balance. These findings highlight a potential mechanism for persistent hyperactivity in neutrophils of periodontal patients. The use of SFN to overcome Nrf2 dysregulation has given a promising insight to restore neutrophil redox balance and reduce hyper-reactivity, although the mechanism of this effect requires further investigation.

## Supporting Information

File S1 Supplementary figures. Figure S1: dHL60 cell viability is not changed with 10µM buthionine sulfoximine (BSO) or 5µM sulphoraphane (SFN) treatments over 16 hours as measured by trypan blue exclusion. Figure S2: dHL60 cell phagocytic capacity is not compromised in the presence of 10µM buthionine sulfoximine (BSO) or 5µM sulphoraphane (SFN) treatments measured by flow cytometry. dHL60 (2×105) cells were incubated with 10µl of pHrodo™ E. coli BioParticles® conjugate (Invitrogen, Paisley, UK) at 370C for 15min. The cells were centrifuged at 350g for 5 min and discarded the supernatant. Cells were washed with PBS two times before analysing by flow cytometer (Beckman coulter, Inc, UK) equipped with a 488 nm argon-ion laser using a 585 nm emission filter. Figure S3: Primary neutrophil viability is not affected by 5µM sulphoraphane (SFN) treatments for 16 hours measured by trypan blue exclusion. Figure S4: Respiratory burst measured by lucigenin dependent chemiluminescence is not quenched by addition of 5mM N-acetyl cysteine (NAC) or 5µM sulphoraphane (SFN) when added at the peak of neutrophil respiratory burst. Figure S5: Sulforaphane (SFN) induced Nrf2 protein expression in primary control neutrophils. Protein expression was detected by western blot. (A) To test the dose–response to SFN, the cells were exposed to the indicated concentrations of SFN for 16hrs. (B) To investigate the time course of Nrf2 and Keap-1 expression; the cells were exposed to 5µM sulforaphane for 1–24 hrs. The upper part shows an original western blot for Nrf2 and Keap-1 (B) expression; the lower part shows results of densitometric analyses normalised to β-actin.
(PDF)
