# Supplementary figures and images for: Sulforaphane Restores Cellular Glutathione Levels and Reduces Chronic Periodontitis Neutrophil Hyperactivity In Vitro
Source: PLoS One. 2013 Jun 24;8(6):e66407. doi: 10.1371/journal.pone.0066407 (PMC3691189; doi:10.1371/journal.pone.0066407)

Figure S1

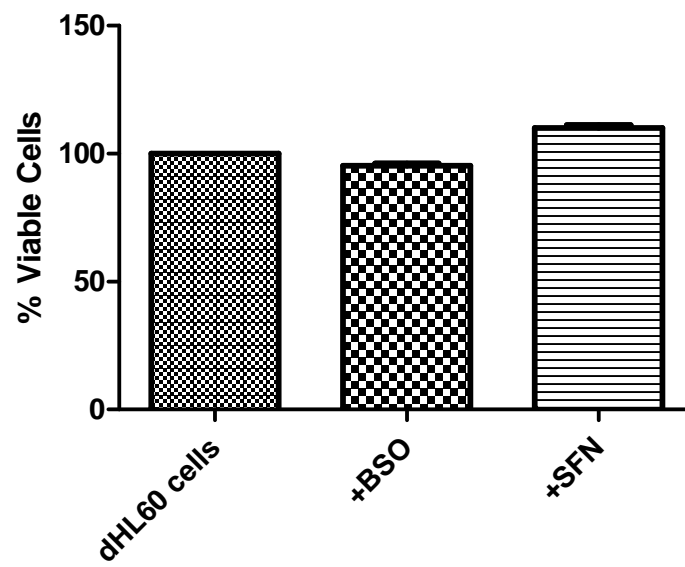

Figure S2

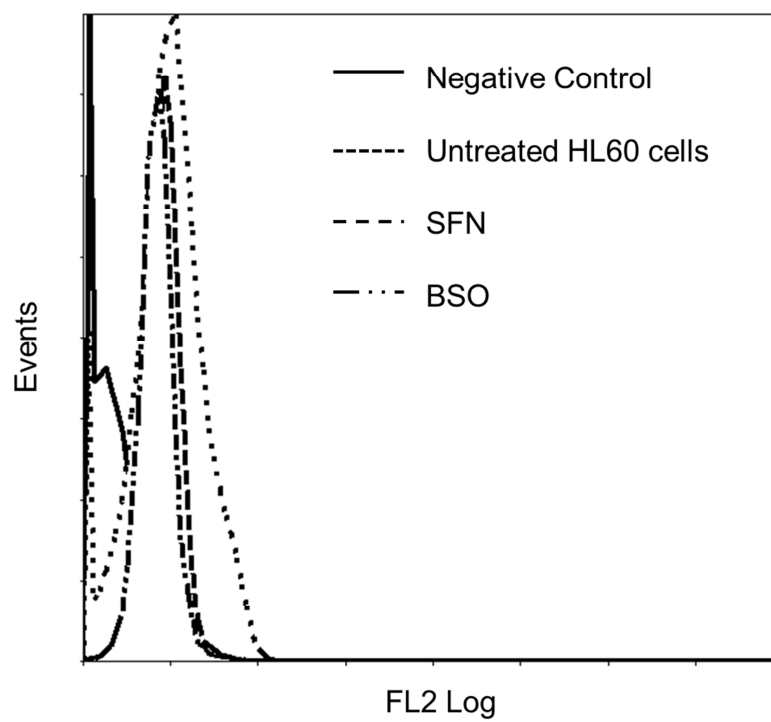

Figure S3

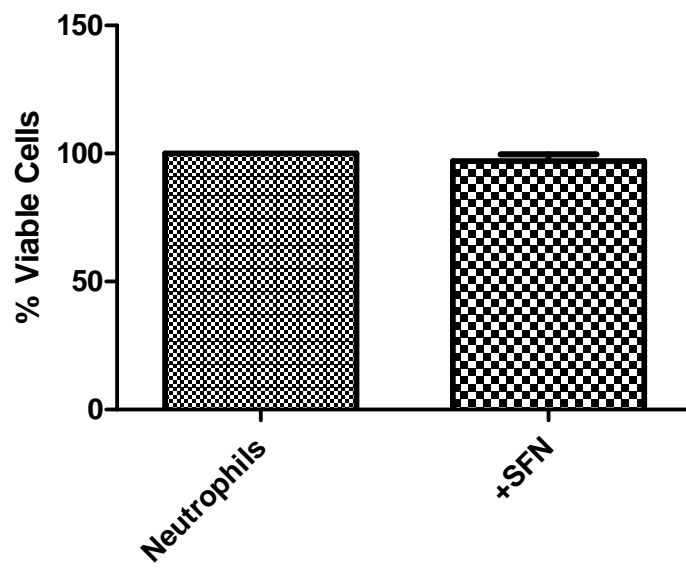

Figure S4

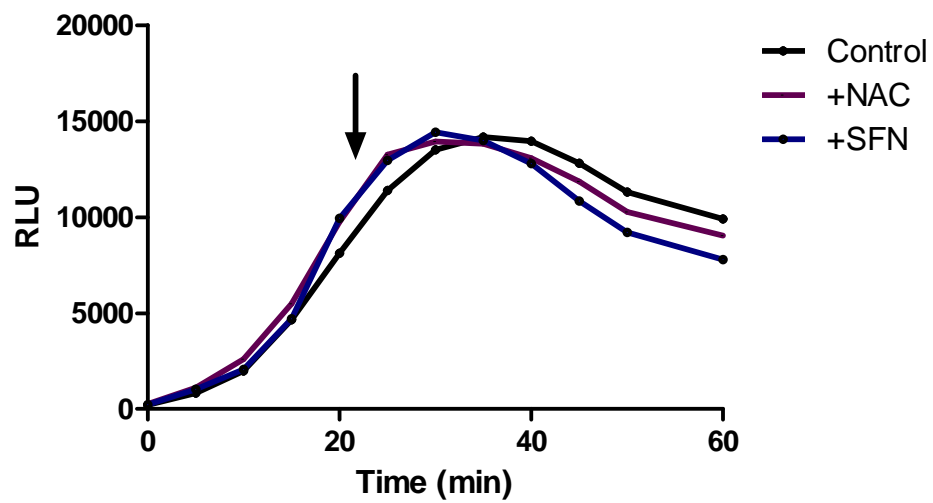

Figure S5

A

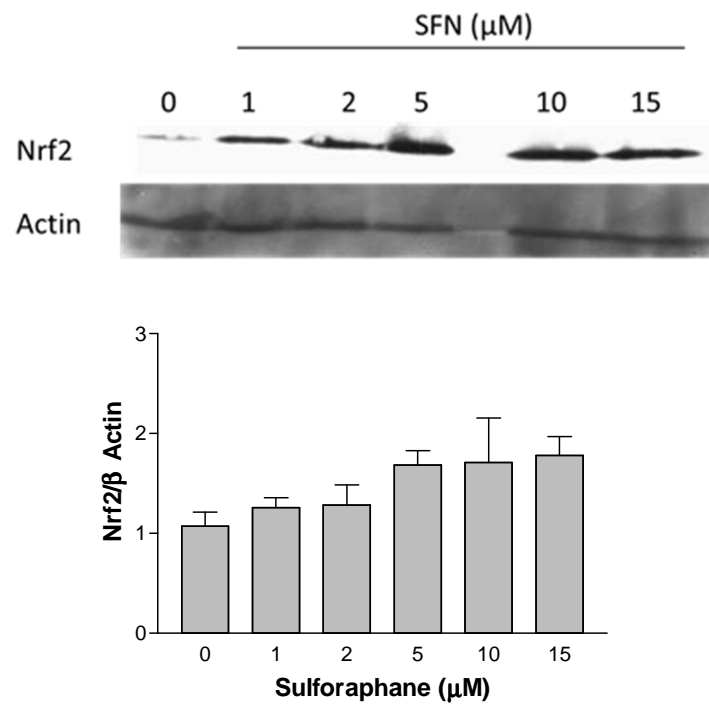

B

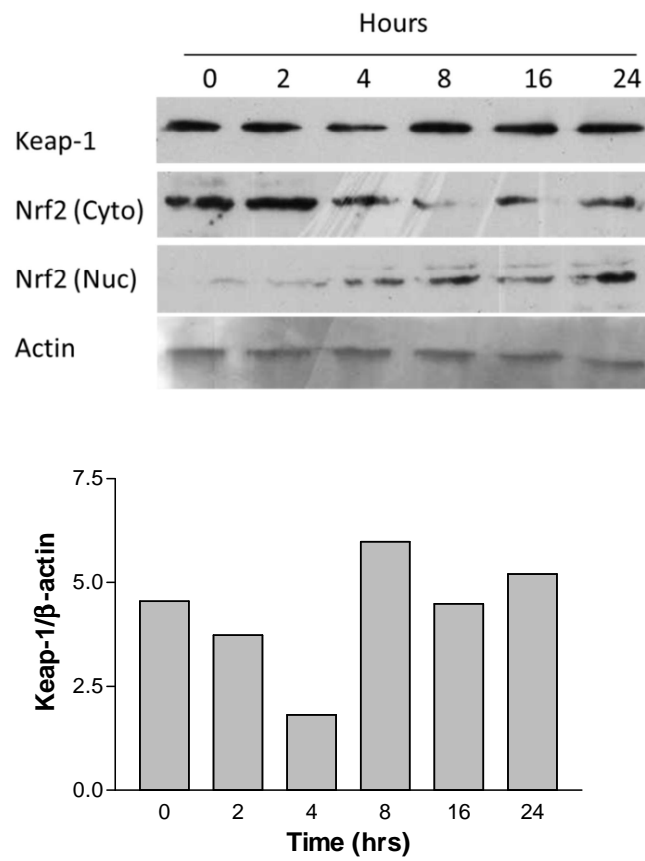

Supplement: File S1 — Figure S1: dHL60 cell viability is not changed with 10µM buthionine sulfoximine (BSO) or 5µM sulphoraphane (SFN) treatments over 16 hours as measured by trypan blue exclusion. Figure S2: dHL60 cell phagocytic capacity is not compromised in the presence of 10µM buthionine sulfoximine (BSO) or 5µM sulphoraphane (SFN) treatments measured by flow cytometry. dHL60 (2×10^5^) cells were incubated with 10µl of pHrodo™ E. coli BioParticles® conjugate (Invitrogen, Paisley, UK) at 370C for 15min. The cells were centrifuged at 350g for 5 min and discarded the supernatant. Cells were washed with PBS two times before analysing by flow cytometer (Beckman coulter, Inc, UK) equipped with a 488 nm argon-ion laser using a 585 nm emission filter. Figure S3: Primary neutrophil viability is not affected by 5µM sulphoraphane (SFN) treatments for 16 hours measured by trypan blue exclusion. Figure S4: Respiratory burst measured by lucigenin dependent chemiluminescence is not quenched by addition of 5mM N-acetyl cysteine (NAC) or 5µM sulphoraphane (SFN) when added at the peak of neutrophil respiratory burst. Figure S5: Sulforaphane (SFN) induced Nrf2 protein expression in primary control neutrophils. Protein expression was detected by western blot. (A) To test the dose–response to SFN, the cells were exposed to the indicated concentrations of SFN for 16hrs. (B) To investigate the time course of Nrf2 and Keap-1 expression; the cells were exposed to 5µM sulforaphane for 1–24 hrs. The upper part shows an original western blot for Nrf2 and Keap-1 (B) expression; the lower part shows results of densitometric analyses normalised to β-actin. (PDF) [file pone.0066407.s001.pdf]
